# Mendelian randomization analysis of smoking, BMI, and nonalcoholic fatty liver disease in European descent populations

**DOI:** 10.1097/MD.0000000000042308

**Published:** 2025-05-02

**Authors:** Lei Ma, Haixing Jiang, Nanfang Qu

**Affiliations:** a The First Affiliated Hospital, Guangxi Medical University, Nanning, Guangxi, China; b The First Affiliated Hospital, Guilin Medical University, Guilin, Guangxi, China.

**Keywords:** body mass index, Mendelian randomization, nonalcoholic fatty liver disease, smoking

## Abstract

Nonalcoholic fatty liver disease (NAFLD) is a chronic liver condition with a steadily increasing prevalence. Evidence indicates that both smoking and obesity are significant risk factors for NAFLD, yet the extent to which smoking influences NAFLD through weight gain remains unclear. This study aimed to dissect the intricate relationship between smoking, body mass index (BMI), and NAFLD using Mendelian randomization (MR) analysis. We leveraged data from 30 genome-wide association studies involving over 1.2 million individuals, from which 123 single nucleotide polymorphisms were selected as instrumental variables for smoking. BMI data were sourced from the Genetic Investigation of Anthropometric Traits (GIANT) consortium, encompassing more than 700,000 individuals, with 521 single nucleotide polymorphisms serving as instrumental variables. NAFLD data were obtained from multiple databases, including the eMERGE Network, UK Biobank, Estonian Biobank, and FinnGen, comprising 8434 cases and 770,180 controls. All participants in this study were of European ancestry. We first applied univariate MR analysis to assess the causal relationship between smoking, NAFLD, and BMI. Subsequently, multivariate MR was used to assess the effect of smoking on NAFLD after adjusting for BMI. The coefficient product method was used to calculate the mediating effect of BMI. Results found that both smoking and high BMI were able to increase the risk of NAFLD, with odds ratios of 1.83 (95% confidence interval [CI]: 1.31–2.55) and 1.58 (95% CI: 1.42–1.77), respectively. BMI mediated 73.3% (95% CI: 62.3%–80.5%) of the effect of smoking on NAFLD. The findings support weight control and the encouragement of smoking cessation, especially in obese populations, as strategies to reduce the risk of NAFLD.

## 1. Introduction

Nonalcoholic fatty liver disease (NAFLD) is a common condition marked by excessive fat accumulation in the liver not related to heavy alcohol use.^[1]^ As a significant health issue globally, NAFLD’s increasing prevalence mirrors the obesity and metabolic syndrome pandemics.^[2,3]^ The disease spectrum includes simple steatosis to nonalcoholic steatohepatitis, with potential progression to fibrosis, cirrhosis, and hepatocellular carcinoma.^[4,5]^

Studies have identified risk factors for NAFLD, such as obesity, insulin resistance, dyslipidemia, and lifestyle choices like diet and exercise.^[6–10]^ Smoking, known for its extensive harm to various organ systems and linkage to cardiovascular and respiratory diseases,^[11,12]^ has recently been scrutinized for its potential role in the development of NAFLD.^[13,14]^ While some research suggests smoking as an independent risk factor for NAFLD, citing toxic effects on liver cells, oxidative stress, and inflammation as mechanisms,^[15–18]^ other studies contest this association.^[19]^

Mendelian randomization (MR), utilizing genetic variants as instrumental variables, offers a means to ascertain causal relationships, circumventing confounding and reverse causality issues common in observational studies.^[20,21]^ Through MR, we can scrutinize the putative causal link between smoking and NAFLD more rigorously.^[22,23]^

Additionally, the potential mediating effect of body mass index (BMI) on the smoking–NAFLD nexus is not well explored. The role of obesity in NAFLD is well-documented,^[24,25]^ and smoking is implicated in weight regulation and metabolic changes.^[26,27]^ Hence, BMI might be an intermediary in the relationship between smoking and NAFLD. Understanding BMI’s mediation could shed light on the mechanisms connecting smoking, body fat, and liver fat accumulation.

This study seeks to determine the causal impact of smoking on NAFLD through MR and to explore BMI’s mediating role in this context. We utilized data from individuals of European descent, offering a consistent genetic framework while providing extrapolative findings to European ancestry populations.

## 2. Methods

### 2.1. Study design

We initially examined the total effect of smoking on NAFLD using univariate MR analysis. We also assessed the influence of smoking on BMI and, in turn, the impact of BMI on NAFLD. Subsequently, we conducted multivariate MR to explore the direct effect of smoking on NAFLD, independent of BMI, and the isolated effect of BMI on NAFLD. We calculated the mediating effect of BMI using the product of coefficients approach, by which we derived the mediation ratio as the mediating effect divided by the total effect of smoking. This study did not require institutional review board approval since all data were sourced from publicly accessible datasets with appropriate consent and ethical clearance.

### 2.2. Data collection

We selected genetic proxies for smoking from a large meta-analysis on tobacco and alcohol use, comprising over 30 genome-wide association studies (GWAS) and involving more than 1.2 million individuals of European ancestry.^[28]^ The smoking index, a validated measure of smoking exposure that integrates the number of cigarettes smoked, the duration of smoking, and the time since quitting, each assigned different weights. Higher scores on this index indicate greater exposure to smoking.^[29,30]^

BMI genetic instruments were sourced from the Genetic Investigation of Anthropometric Traits consortium’s GWAS meta-analysis, which included approximately 700,000 European individuals. Here, 1 standard deviation change equated to a 4.8 kg/m^2^ difference in BMI.^[31]^

NAFLD-related GWAS data were obtained from a comprehensive meta-analysis encompassing the eMERGE Network, UK Biobank, Estonian Biobank, and FinnGen.^[32]^ We included 8434 cases and 770,180 controls, all of European descent. NAFLD was diagnosed using specific electronic health record codes. Exclusion criteria were aligned with the American Association for the Study of Liver Diseases guidelines and included various liver-related disorders.

There was a certain degree of overlap among the 3 study populations, primarily due to the shared use of partial data from the United Kingdom Biobank (Supplementary Table 1, Supplemental Digital Content, https://links.lww.com/MD/O839). The overlap rates were 10.38% between the smoking and NAFLD datasets, 16.18% between the smoking and BMI datasets, and 17.80% between the BMI and NAFLD datasets. We conducted separate MR analyses for each dataset, following the approach by Burgess et al^[33]^ (https://sb452.shinyapps.io/overlap/), to calculate bias and type 1 error rates. The results showed that the overlapping samples did not increase the type 1 error rate in the MR analyses. However, in the MR analysis of the effect of BMI on NAFLD, there was a slight increase in bias of 0.1% (Supplementary Table 2, Supplemental Digital Content, https://links.lww.com/MD/O839).

### 2.3. Genetic instrument selection

The MR framework relies on 3 core assumptions: genetic instruments are associated with the exposure, they are not related to confounders, and they influence the outcome exclusively through the exposure.^[34,35]^ We conducted a stepwise screening of genetic instruments in line with these principles. Initially, GWAS databases were mined for single nucleotide polymorphisms (SNPs) significantly associated with exposure, with a *P* value threshold of 5 × 10^−8^. Next, we removed SNPs in linkage disequilibrium, utilizing a 10 Mb window and an *R*^2^ threshold of <0.001. From the remaining SNPs, we excluded those associated with the outcome at *P* < 5 × 10^-6^, as well as palindromic SNPs and those linked to potential confounders. An F-statistic was calculated to assess the strength of the genetic instruments, with an *F* > 10 indicating suitability for MR analysis.^[34,36]^

### 2.4. Statistical methods for MR

For MR analysis, we employed a suite of statistical methods. In univariate MR, we utilized inverse variance weighted (IVW), MR-Egger, weighted median, simple mode, and weighted mode. For multivariate MR, we applied IVW, MR-Egger, and weighted median. IVW results served as the primary findings, assuming all SNPs were valid instruments.^[37]^ Heterogeneity was assessed using Cochran Q statistic in both IVW and MR-Egger methods. Multiplicity issues were addressed with the MR-Egger intercept test and the global test in the MRPRESSO package. Results were visualized using leave-one-out analysis, forest plots, funnel plots, and scatter plots. To avoid bias introduced by heterogeneity, we used the IVW random-effects model.

All analyses were conducted using the “TwoSampleMR” and “MendelianRandomization” R packages (version 4.3.1, R Foundation for Statistical Computing, Vienna, Austria). We completed 3 univariate and 1 multivariate MR analyses. Statistical significance was determined using Bonferroni correction, with a threshold of *P* < .0125, adjusting for the 4 tests conducted.

## 3. Results

### 3.1. Instrumental variables for study

We extracted a total of 123 instrumental variables for smoking, which accounted for 0.18% of the variance in smoking behaviors, as indicated by an F-statistic of 17.8 (Supplementary Table 3, Supplemental Digital Content, https://links.lww.com/MD/O839). For BMI, 521 SNPs were identified, explaining 2.13% of the variance with a robust F-statistic of 29.2 (Supplementary Table 4, Supplemental Digital Content, https://links.lww.com/MD/O839), thereby minimizing the risk of weak instrument bias (Table 1).

**Table 1:** Summary of GWAS information for study included.

Study	Authors	PMID	Sample size	nSNP	*R*^2^ (%)	*F*
Smoking	Liu et al^[28]^	30643251	1,200,000	123	0.18	17.8
BMI	Yengo et al^[31]^	30124842	700,000	521	2.13	29.2
NAFLD	Ghodsian et al^[32]^	34841290	778,614 (8434 cases)	/	/	/

BMI = body mass index, *F* = F-statistic, GWAS = genome-wide association studies, NAFLD = nonalcoholic fatty liver disease, *R*^2^ = explained variance, SNP = single nucleotide polymorphism, / = none.

### 3.2. Univariate MR results

**Figure 1.**
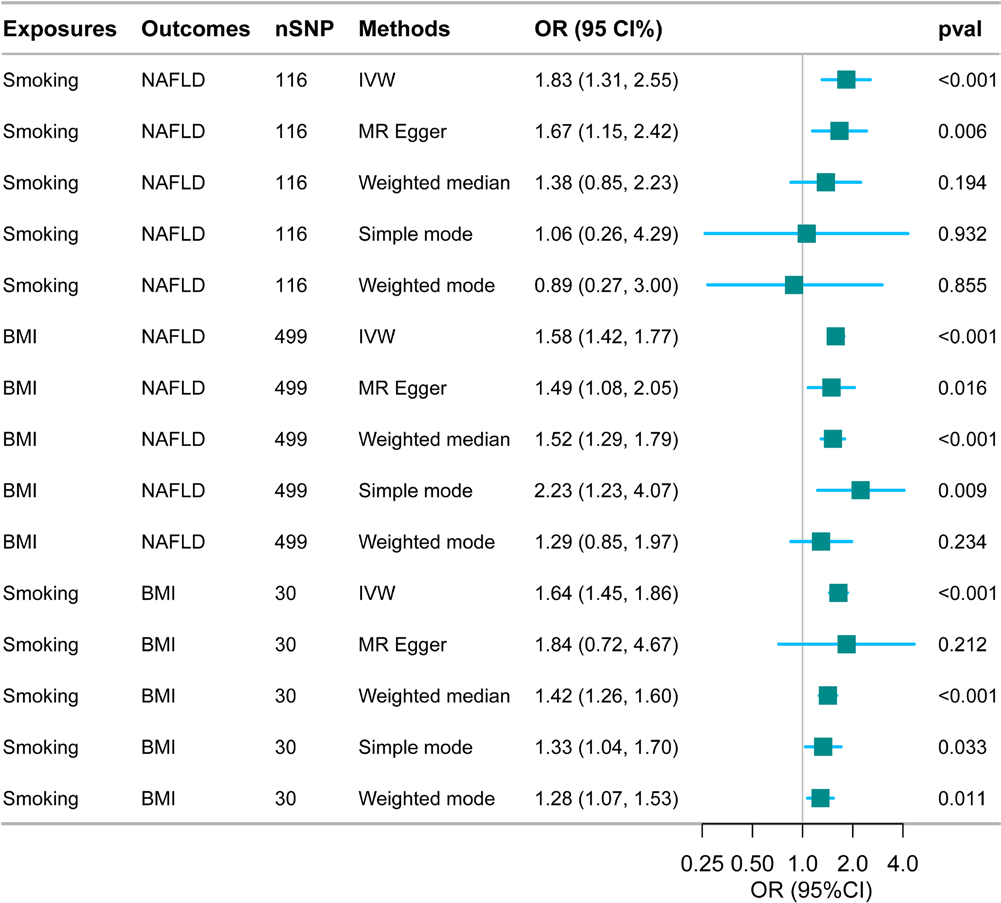
Univariate Mendelian randomization analysis. BMI = body mass index, CI = confidence interval, IVW = inverse variance weighted, MR = Mendelian randomization, NAFLD = nonalcoholic fatty liver disease, OR = odd ratio, SNP = single nucleotide polymorphism.

The univariate MR analysis revealed a significant causal relationship between smoking, BMI, and the risk of NAFLD. The odds ratio (OR) for the impact of smoking on NAFLD was 1.83 (95% confidence interval [CI]: 1.3–2.55). Additionally, a notable association between BMI and NAFLD was observed, with an OR of 1.58 (95% CI: 1.42–1.77) for BMI’s effect on NAFLD. The analysis also confirmed a significant link between smoking and BMI, yielding an OR of 1.64 (95% CI: 1.45–1.86) for the effect of smoking on BMI. These findings are illustrated in Figure 1.

### 3.3. Multivariate MR results

**Figure 2.**
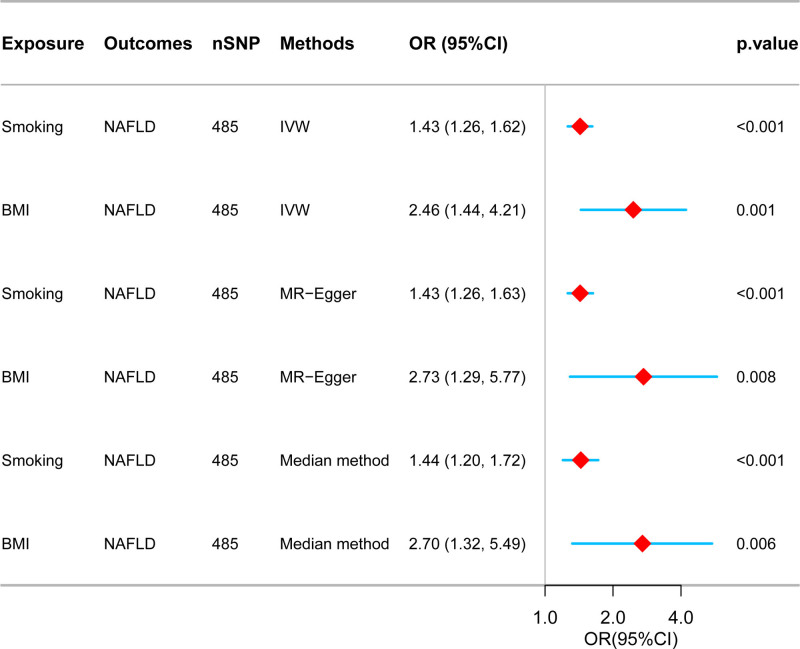
Multivariate Mendelian randomization analysis. BMI = body mass index, CI = confidence interval, IVW = inverse variance weighted, MR = Mendelian randomization, NAFLD = nonalcoholic fatty liver disease, OR = odd ratio, SNP = single nucleotide polymorphism.

Upon adjusting for smoking, BMI retained a significant association with NAFLD in the multivariate analysis. The OR for the independent effect of BMI on NAFLD was 2.46 (95% CI: 1.44–4.21), illustrating a substantial independent influence on NAFLD risk. Additionally, the direct effect of smoking on NAFLD, independent of BMI, was significant, with an OR of 1.43 (95% CI: 1.26–1.62), suggesting that smoking directly contributes to the risk of NAFLD. These findings are illustrated in Figure 2.

### 3.4. Mediating effect of BMI

**Figure 3.**
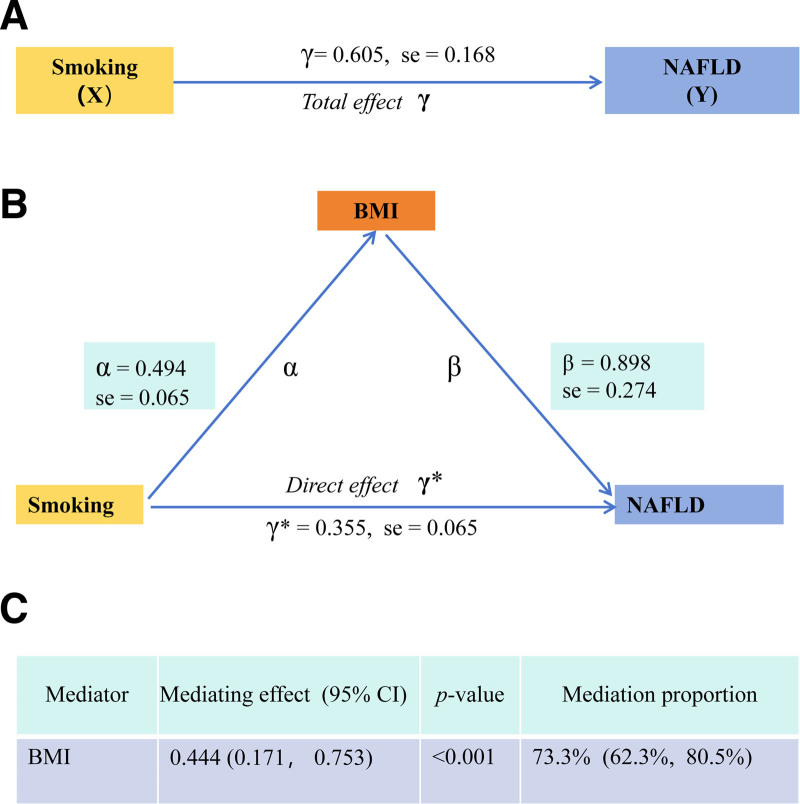
Mediating effects model for BMI. (A) The total effect of smoking on NAFLD. (B) The effect of smoking on BMI, the direct effect of smoking on NAFLD, and the effect of BMI on NAFLD. (C) The proportion of the mediating effect of BMI mediating the effect of smoking on NAFLD. BMI = body mass index, CI = confidence interval, NAFLD = nonalcoholic fatty liver disease.

The total effect of smoking on NAFLD is significant (Fig. 3A). Then, we assessed BMI’s mediating role in the smoking–NAFLD relationship using the product of coefficients method. The mediating effect attributed to BMI was significant, with *β* = 0.444 (95% CI: 0.171–0.753, *P* < .001), as illustrated in Figure 3B. This mediating effect represented 73.3% (95% CI: 62.3%–80.5%) of smoking’s total effect on NAFLD (Fig. 3C), indicating that a substantial proportion of the smoking–NAFLD association is mediated through BMI.

### 3.5. Sensitivity analyses

Heterogeneity was detected in the MR analyses concerning the effects of BMI on NAFLD and smoking on BMI (Supplementary Table 5, Supplemental Digital Content, https://links.lww.com/MD/O839). No evidence of pleiotropy was found in any of the MR analyses (Supplementary Tables 6 and 7, Supplemental Digital Content, https://links.lww.com/MD/O839). To investigate any single SNP’s substantial impact on the results, we conducted a reanalysis excluding outliers using the MRPRESSO package. This included generating scatter plots (Supplemental File 8, Supplemental Digital Content, https://links.lww.com/MD/O839), funnel plots (Supplemental File 9, Supplemental Digital Content, https://links.lww.com/MD/O839), and leave-one-out plots (Supplemental File 10, Supplemental Digital Content, https://links.lww.com/MD/O839). Despite the thorough investigation, no individual SNP was found to significantly alter the results. Additionally, in the supplementary method results, including MR-Egger, weighted median, weighted mode, and simple mode, the 95% CIs were broader, leading to a loss of statistical significance for some estimates. Nevertheless, the causal direction remained largely consistent with the IVW method (Figs. 1 and 2).

## 4. Discussion

NAFLD is a prevalent and complex metabolic disorder characterized by hepatic fat accumulation. It is closely associated with obesity, insulin resistance, and other metabolic abnormalities.^[1,38,39]^ Smoking has been implicated as a potential risk factor for NAFLD, though the underlying mechanisms and the mediating role of BMI in this relationship remain to be fully elucidated.^[13]^ Our study conducted a MR analysis to investigate the causal effect of smoking on NAFLD and to explore BMI’s mediating role. Our results provide novel insights into the interplay between smoking, BMI, and NAFLD, informing potential prevention and management strategies.

Our univariate MR analysis confirmed a significant positive association between smoking, BMI, and the risk of NAFLD. This aligns with previous reports, such as the study by Yuan et al,^[10]^ which used MR to investigate the impact of 5 lifestyle factors and 9 metabolic traits on NAFLD development, identifying smoking and BMI as independent risk factors for NAFLD. Our study further explored BMI’s role as a mediator in the relationship between smoking and NAFLD. These findings are consistent with epidemiological evidence suggesting a higher prevalence of NAFLD among smokers and individuals with elevated BMI.^[40–42]^ The detrimental effects of smoking on liver health are multifaceted, including oxidative stress, inflammation, impaired lipid metabolism, and changes in gut microbiota.^[43,44]^ Conversely, excess adiposity can lead to increased release of free fatty acids from adipose tissue, promoting liver steatosis, inflammation, and fibrosis.^[45,46]^

Moreover, our analysis revealed a significant correlation between smoking and BMI, with an estimated OR of 1.64 (95% CI: 1.45–1.86), indicating that smoking is associated with an increased BMI, which may suggest a potential influence of smoking on weight gain or adiposity. The relationship between smoking and BMI is indeed complex. Some studies have shown that smoking can affect nutrient absorption, potentially leading to a lower BMI,^[47]^ while others have found that smoking can increase BMI. Both observational studies^[48]^ and MR analyses^[49]^ have reported these mixed results, suggesting that the relationship between smoking and BMI may be nonlinear and warrants further investigation. The complexity of this relationship involves alterations in energy expenditure, appetite regulation, and lipid metabolism, which can contribute to weight gain.^[50,51]^ Additionally, weight gain often occurs following smoking cessation, further complicating the association between smoking and BMI.^[52]^

Delving deeper into the interaction between smoking, BMI, and NAFLD through multivariate MR analyses, we found that smoking retained a significant relationship with NAFLD after adjusting for BMI, with an estimated OR of 1.43 (95% CI: 1.26–1.62). This indicates that smoking may impact NAFLD risk independently of its effect on BMI. Potential mechanisms for smoking’s direct effect on NAFLD include oxidative stress, inflammation, and changes in hepatic lipid metabolism.^[53,54]^ Future research is needed to further clarify these pathways. A critical aspect of our study was evaluating the mediating role of BMI in the smoking–NAFLD nexus, where we determined that BMI mediated 73.3% (95% CI: 62.3%–80.5%) of smoking’s total effect on NAFLD. This finding highlights BMI’s pivotal role in the pathway from smoking to NAFLD. Given the global prevalence of smoking and obesity, interventions that address both could significantly reduce NAFLD’s burden.

Our study’s limitations warrant mention. First, the validity of our MR analysis hinges on the genetic variants used as instruments satisfying the instrumental variable assumptions. Despite selecting variants strongly associated with smoking and BMI, assumptions violations could introduce bias to our estimates. Second, while focusing on the nexus between smoking, BMI, and NAFLD, other contributing factors, such as diet, physical activity, and genetic predisposition, should be considered in future studies for a holistic understanding of NAFLD etiology. Third, our reliance on summary-level data from GWAS limited our ability to assess potential effect modifiers or interactions among smoking, BMI, and NAFLD. Further research incorporating individual-level data and examining gene–environment interactions would be beneficial. Finally, while MR can establish causal relationships, it does not elucidate the biological mechanisms involved. Experimental studies and functional investigations are necessary to uncover how smoking, BMI, and NAFLD interrelate.

In conclusion, our study presents strong evidence for a causal link between smoking and an elevated risk of NAFLD. We have shown that smoking not only contributes to an increased risk of NAFLD through BMI but also has a direct effect independent of BMI. These findings emphasize the need to tackle both smoking and obesity as key modifiable risk factors in NAFLD prevention and management.

## Acknowledgments

The study was based on summary statistics provided by the Tobacco and Alcohol Consumption, Anthropometric Traits Consortium, eMERGE Network, UK Biobank, Estonian Biobank, and FinnGen. We thank all investigators and consortiums for sharing valuable summary data.

## Author contributions

**Conceptualization:** Lei Ma, Haixing Jiang, Nanfang Qu.

**Data curation:** Lei Ma, Nanfang Qu.

**Writing – original draft:** Lei Ma, Nanfang Qu.

**Writing – review & editing:** Lei Ma, Haixing Jiang.

**Validation:** Haixing Jiang.

**Visualization:** Haixing Jiang.

**Formal analysis:** Nanfang Qu.

**Methodology:** Nanfang Qu.

## Supplementary Material


