# Supplementary material for: Mendelian randomization analysis of smoking, BMI, and nonalcoholic fatty liver disease in European descent populations
Source: Medicine (Baltimore). 2025 May 2;104(18):e42308. doi: 10.1097/MD.0000000000042308 (PMC12055159; doi:10.1097/MD.0000000000042308)
Supplement: Supplementary file 1 [file medi-104-e42308-s001.pdf]

Supplementary Table 1. Smoking, BMI and NAFLD study cohorts summary

Smoking cohorts		BMI cohorts		NAFLD cohorts		
Study	N	Study	N	Study	cases	controls
23andMe[a]	78,437	ACTG	2,648	eMERGE	1,106	8,571
ALSPAC	4,691	AE	2,512	FinnGen	651	176,248
ARIC	5,559	ASCOT	3,868	UKB	2,558	395,241
BLS	546	BLSA	848	Estonian Biobank	4,119	190,120
CADD	775	B-PROOF	2919			
COGEND	1,952	DESIR	731			
COPDGene	6,613	DNBC	1937			
deCODE	40,314	EGCUT-370	866			
EGCUT	5492	EGCUT-OMNI	1356			
FHS	3,025	ERF	2726			
FinnTwin	509	FamHS	1486			
GFG	855	Health ABC	1655			
Harvard	4,756	HERITAGE	500			
HRS	5,585	HYPERGENES	1934			
HUNT	35,311	HYPERGENES	2124			
MESA	1,183	InCHIANTI	1210			
METSIM	1,500	IPM Mount	3069			
NAG-FIN	1,704	LifeLines	9480			
NTR	2,955	LLS	2415			
QIMR	4,193	LOLIPOP_EW610	945			
SardiNIA	2,057	LOLIPOP_EWA	878			
UKB[c]	124,590	LOLIPOP_EWP	1006			
WHI	8,825	NELSON	3082			
		PLCO2 cases	3003			
		PLCO2 controls	1216			
		PREVEND	3920			
		PROCARDIS	13000			
		PROSPER	5.784			
		QFS	951			
		QIMR	11930			
		RISC	1566			
		SHIP-TREND	997			
		TRAILS	1491			
		TWINGENE	9836			
		UKB	450000			

Supplementary Table 2. Probability of IV bias and type I error in MR analysis

Exposures	Outcomes	Overlap proportion	Bias	Type 1 error rate
Smoking	NAFLD	10.38 %	0	0.05
Smoking	BMI	16.18 %	0	0.05
BMI	NAFLD	17.80 %	0.001	0.05

BMI, body mass index; NAFLD, non-alcoholic fatty liver disease;

Supplementary Table 3. Instrumental variables for Smoking

NO.	SNP	EA	OA	BETA	EAF	SE	pval
1	rs11768481	C	A	0.00901238	0.666168	0.00147464	9.90E-10
2	rs17309874	G	A	-0.0112899	0.740357	0.00158234	9.70E-13
3	rs359243	T	C	-0.00871975	0.392886	0.00142539	9.50E-10
4	rs986391	G	A	0.0111388	0.366555	0.0014379	9.40E-15
5	rs3811038	T	C	-0.00954466	0.723852	0.00155745	8.90E-10
6	rs35343344	C	A	0.00918144	0.732713	0.00159594	8.80E-09
7	rs549845	G	A	0.0112613	0.301161	0.00150857	8.30E-14
8	rs6935954	A	G	0.0095817	0.421108	0.00140192	8.20E-12
9	rs13296519	G	T	-0.00970128	0.606385	0.00141888	8.10E-12
10	rs62135536	C	T	0.0243444	0.968256	0.00396165	8.00E-10
11	rs12481282	G	C	-0.00894356	0.722343	0.00154935	7.80E-09
12	rs6962772	A	G	0.011064	0.845551	0.00191645	7.80E-09
13	rs12623702	A	G	-0.00977238	0.613375	0.00142787	7.70E-12
14	rs9919670	G	A	-0.0152438	0.612185	0.00142114	7.60E-27
15	rs7569203	A	C	-0.0107595	0.688725	0.0015002	7.40E-13
16	rs245774	A	G	-0.00901601	0.271691	0.00155972	7.40E-09
17	rs1221148	C	G	0.00916504	0.587062	0.00140711	7.30E-11
18	rs7807019	A	G	-0.0104211	0.540327	0.00139062	6.70E-14
19	rs624833	T	G	0.00928567	0.694697	0.00150361	6.60E-10
20	rs7297175	T	C	-0.00811649	0.431355	0.0013992	6.60E-09
21	rs4473348	A	T	-0.0104317	0.249978	0.00159675	6.40E-11
22	rs329120	C	T	0.00965704	0.580672	0.00140509	6.30E-12
23	rs889398	C	T	0.0092407	0.588005	0.00141367	6.30E-11
24	rs2838834	C	T	-0.00936489	0.699459	0.00151488	6.30E-10
25	rs10282292	C	T	0.00896219	0.361838	0.0014469	5.90E-10
26	rs7528604	G	A	0.00965269	0.565613	0.00140141	5.70E-12
27	rs17553262	A	C	-0.0127323	0.884605	0.00218148	5.30E-09
28	rs62155874	A	G	-0.0169073	0.87336	0.00208546	5.20E-16
29	rs7553348	G	A	0.00963399	0.437656	0.00139643	5.20E-12
30	rs7039819	G	A	0.00873433	0.427307	0.00140501	5.10E-10
31	rs10879871	T	G	-0.00957988	0.343436	0.00145803	5.00E-11
32	rs11948770	T	C	-0.0102364	0.768248	0.00164524	4.90E-10
33	rs2867112	T	G	0.0147832	0.834596	0.00188747	4.80E-15
34	rs35175834	G	A	-0.0164032	0.78844	0.00169847	4.60E-22
35	rs13009008	A	G	0.00863272	0.327713	0.00147329	4.60E-09
36	rs4543592	T	C	-0.00866243	0.519912	0.0013891	4.50E-10
37	rs6598539	T	C	-0.00815056	0.488672	0.00138941	4.50E-09
38	rs6957896	C	T	-0.00758434	0.503261	0.00138695	4.50E-08
39	rs1246265	T	C	-0.00886526	0.30456	0.00150907	4.20E-09
40	rs4957528	A	C	-0.0101225	0.208497	0.00172212	4.20E-09
41	rs61796681	A	T	-0.0134163	0.912365	0.00244752	4.20E-08

42	rs62098013	G	A	-0.00856663	0.63991	0.00145676	4.10E-09
43	rs1931263	G	T	-0.00760874	0.510321	0.0013858	4.00E-08
44	rs57611503	G	A	0.00774344	0.484578	0.00141026	4.00E-08
45	rs202645	A	G	-0.0101598	0.202939	0.00172497	3.90E-09
46	rs112282219	G	A	-0.0231662	0.958949	0.00350418	3.80E-11
47	rs72674867	A	T	0.00898766	0.764568	0.00163396	3.80E-08
48	rs6119897	G	A	-0.0128038	0.761716	0.00162704	3.60E-15
49	rs12708665	A	G	-0.0090871	0.284632	0.00153886	3.50E-09
50	rs2894808	T	A	-0.0152983	0.922121	0.0025912	3.50E-09
51	rs2254710	C	A	0.00899636	0.236356	0.00163085	3.50E-08
52	rs421983	T	C	0.00870743	0.519028	0.00138561	3.30E-10
53	rs7333559	G	A	0.01074	0.211711	0.00170716	3.20E-10
54	rs2678670	A	T	0.00873079	0.485528	0.00138756	3.10E-10
55	rs9842947	C	T	-0.00877189	0.326165	0.00148073	3.10E-09
56	rs12967855	A	G	0.0081887	0.331225	0.00147934	3.10E-08
57	rs113382419	C	A	-0.0282339	0.889325	0.00221376	3.00E-37
58	rs10922907	A	T	0.0101748	0.45105	0.00139495	3.00E-13
59	rs1922018	C	T	0.0100328	0.364422	0.00143771	3.00E-12
60	rs60952428	T	C	0.0134109	0.909149	0.00241933	3.00E-08
61	rs147412694	G	A	-0.0115726	0.850009	0.00194865	2.90E-09
62	rs4814873	C	T	0.00971214	0.76659	0.00163637	2.90E-09
63	rs12202536	A	G	-0.00823391	0.512754	0.00138601	2.80E-09
64	rs1193237	G	C	-0.00776138	0.439176	0.00139716	2.80E-08
65	rs2401924	G	C	0.0105718	0.501955	0.00138906	2.70E-14
66	rs860326	C	T	0.00833808	0.427696	0.0014023	2.70E-09
67	rs7077678	C	T	0.00855025	0.623429	0.00143575	2.60E-09
68	rs28485305	C	T	0.00800666	0.631482	0.00143887	2.60E-08
69	rs4731925	C	T	-0.00829239	0.315607	0.00149022	2.60E-08
70	rs3742365	T	C	-0.0107929	0.595136	0.0014159	2.50E-14
71	rs13153393	A	G	-0.0137451	0.883937	0.00217329	2.50E-10
72	rs3769949	T	A	-0.00825646	0.527845	0.00138563	2.50E-09
73	rs7155595	A	C	-0.00885162	0.674282	0.00148508	2.50E-09
74	rs6011779	C	T	0.0191152	0.191239	0.00176364	2.30E-27
75	rs11255908	T	G	-0.0100713	0.743485	0.00158809	2.30E-10
76	rs7766610	C	A	0.0125839	0.182634	0.00179283	2.20E-12
77	rs2890772	G	T	-0.0136979	0.413181	0.00140672	2.10E-22
78	rs10052591	T	C	0.00839979	0.573358	0.00140216	2.10E-09
79	rs10823968	A	T	0.00813544	0.632969	0.00145258	2.10E-08
80	rs10918701	G	A	0.00802389	0.372247	0.00143191	2.10E-08
81	rs74086911	G	A	0.0148021	0.925459	0.00264197	2.10E-08
82	rs11210229	A	G	0.011702	0.383666	0.00142273	2.00E-16
83	rs10226228	A	G	-0.0114099	0.62964	0.00143702	2.00E-15
84	rs11861214	G	T	0.00945079	0.78364	0.00168455	2.00E-08
85	rs12831617	C	T	-0.00918459	0.764305	0.00163314	1.90E-08

86	rs8042849	C	T	0.0192164	0.34229	0.00146183	1.80E-39
87	rs8614	C	A	-0.0114612	0.817489	0.00179679	1.80E-10
88	rs13016665	C	A	-0.00848745	0.576725	0.0014118	1.80E-09
89	rs369230	G	T	-0.00908882	0.307565	0.001511	1.80E-09
90	rs136233	A	G	-0.00995614	0.809084	0.00176918	1.80E-08
91	rs17576594	G	A	0.0109502	0.7235	0.00155159	1.70E-12
92	rs4949465	T	C	-0.0116055	0.869552	0.00205788	1.70E-08
93	rs62175972	T	C	0.0217461	0.965939	0.00385678	1.70E-08
94	rs72678864	G	A	0.0123829	0.828591	0.0018387	1.60E-11
95	rs71627581	G	A	0.0132566	0.888811	0.00219871	1.60E-09
96	rs6741228	T	C	0.00793282	0.433309	0.00140363	1.60E-08
97	rs1933270	T	G	0.00921831	0.363726	0.00143808	1.50E-10
98	rs4571506	C	T	0.00787701	0.539523	0.00139172	1.50E-08
99	rs732083	G	A	0.00834823	0.333362	0.00147311	1.50E-08
100	rs73220544	A	C	-0.0108189	0.842295	0.0019126	1.50E-08
101	rs12244388	G	A	-0.0132503	0.661124	0.00146413	1.40E-19
102	rs4391802	A	G	0.0103191	0.707464	0.00152789	1.40E-11
103	rs71367545	G	A	-0.0103197	0.790533	0.0017038	1.40E-09
104	rs1050847	C	T	0.00797046	0.425845	0.00140503	1.40E-08
105	rs6778080	T	C	0.0111141	0.267372	0.00156621	1.30E-12
106	rs8042134	T	G	-0.00994136	0.541083	0.0014011	1.30E-12
107	rs2675638	G	A	0.00849857	0.58057	0.00140017	1.30E-09
108	rs75742406	G	A	0.00961948	0.738854	0.00158486	1.30E-09
109	rs348809	A	G	-0.00828381	0.347637	0.00145602	1.30E-08
110	rs11783093	C	T	0.01571	0.838811	0.00189655	1.20E-16
111	rs326341	G	A	0.00943522	0.524943	0.00139247	1.20E-11
112	rs35169606	T	G	0.00877627	0.612204	0.00144385	1.20E-09
113	rs67596067	G	A	-0.00887666	0.648812	0.00145843	1.20E-09
114	rs6779302	G	T	-0.00875253	0.632921	0.0014392	1.20E-09
115	rs34866095	A	G	-0.00857198	0.686187	0.00150558	1.20E-08
116	rs7519626	C	T	0.00842015	0.323676	0.00147893	1.20E-08
117	rs9435340	T	A	0.00834773	0.344234	0.00146424	1.20E-08
118	rs3896224	A	G	0.00962696	0.585286	0.00141792	1.10E-11
119	rs4671357	T	C	-0.00944175	0.518819	0.00138991	1.10E-11
120	rs317021	T	A	-0.011567	0.814287	0.00179087	1.10E-10
121	rs2062882	G	A	-0.0081103	0.586782	0.00141998	1.10E-08
122	rs775758	A	T	0.00801379	0.432859	0.00140266	1.10E-08
123	rs6562474	C	G	0.00836914	0.650606	0.0014611	1.00E-08

SNP:Single nucleotide polymorphism; EA: effect allele; OA: other allele; EAF: expected average frequency;SE: standard error.

Supplementary Table 4. Instrumental variables for BMI

NO.	SNP	EA	OA	BETA	EAF	SE	pval
1	rs9926784	T	C	0.0258	0.8178	0.0021	9.90E-35
2	rs12602912	T	C	0.0176	0.2048	0.0021	9.90E-18
3	rs4012234	T	G	-0.0141	0.4076	0.0018	9.90E-16
4	rs1266874	A	G	-0.014	0.6442	0.0018	9.80E-15
5	rs577525	T	C	-0.0166	0.4324	0.0017	9.70E-22
6	rs12652212	A	G	-0.0123	0.5786	0.0017	9.70E-14
7	rs2612576	A	T	-0.011	0.2851	0.0019	9.70E-09
8	rs12429545	A	G	0.0316	0.1248	0.0025	9.60E-38
9	rs10478110	A	C	-0.01	0.5652	0.0017	9.60E-09
10	rs12041258	T	C	0.0146	0.7713	0.002	9.50E-13
11	rs7724675	A	G	-0.0119	0.2238	0.0021	9.50E-09
12	rs1477199	A	G	-0.0228	0.8549	0.0024	9.40E-22
13	rs2285178	T	C	-0.0112	0.6891	0.0019	9.40E-09
14	rs987237	A	G	-0.0409	0.8197	0.0021	9.30E-84
15	rs7551507	T	C	-0.0184	0.5633	0.0016	9.30E-30
16	rs10795422	A	G	-0.0139	0.3095	0.0019	9.30E-14
17	rs7249149	A	G	0.0124	0.7256	0.0019	9.30E-11
18	rs1320903	A	G	0.0216	0.3174	0.0018	9.20E-32
19	rs10824345	T	C	0.0105	0.5056	0.0017	9.20E-10
20	rs6545714	A	G	-0.0191	0.6139	0.0017	9.10E-31
21	rs1528435	T	C	0.0164	0.6331	0.0017	9.10E-23
22	rs7730004	T	C	0.0148	0.6693	0.0018	9.10E-16
23	rs12888545	A	G	-0.0136	0.7481	0.002	9.10E-12
24	rs10827649	A	G	-0.0094	0.5723	0.0016	9.10E-09
25	rs329651	T	G	0.0164	0.8055	0.0021	9.00E-15
26	rs1784460	A	T	0.0132	0.4035	0.0018	9.00E-14
27	rs1371108	A	C	0.0119	0.3247	0.0018	9.00E-11
28	rs7983065	T	C	-0.0148	0.4503	0.0017	8.90E-18
29	rs4937870	A	G	0.0109	0.6828	0.0019	8.80E-09
30	rs10867256	T	C	-0.0118	0.553	0.0017	8.70E-12
31	rs1937684	A	T	0.0112	0.6592	0.0018	8.70E-10
32	rs7694732	A	G	0.0099	0.5622	0.0017	8.70E-09
33	rs6593688	A	G	-0.0137	0.6267	0.0018	8.60E-15
34	rs11614340	T	C	-0.0133	0.6908	0.0019	8.50E-13
35	rs1937433	C	G	-0.0124	0.4642	0.0017	8.50E-13
36	rs7488867	T	C	-0.0204	0.2639	0.002	8.40E-24
37	rs8097672	A	T	-0.02	0.8472	0.0025	8.40E-16
38	rs3749897	T	C	0.0122	0.4172	0.0018	8.40E-12
39	rs2192158	A	G	0.0129	0.4601	0.0017	8.30E-14
40	rs2242189	T	C	0.0135	0.6342	0.0018	8.30E-14
41	rs4968656	A	G	-0.0116	0.6784	0.0019	8.20E-10
42	rs12448257	A	G	0.0184	0.218	0.002	8.10E-20

43	rs11066188	A	G	-0.012	0.4181	0.0017	8.10E-13
44	rs11615578	T	C	0.013	0.2474	0.002	8.10E-11
45	rs11856579	A	G	-0.0141	0.2654	0.002	8.00E-13
46	rs3731695	T	C	-0.0116	0.4418	0.0016	7.90E-13
47	rs9375702	T	C	-0.0115	0.705	0.0019	7.90E-10
48	rs17535082	T	C	-0.0171	0.0939	0.003	7.80E-09
49	rs10492229	T	C	0.0142	0.2268	0.0019	7.70E-14
50	rs11185111	A	G	-0.0129	0.3042	0.0019	7.70E-12
51	rs12334877	A	G	-0.0144	0.198	0.0022	7.70E-11
52	rs16903285	T	C	-0.0331	0.8593	0.0026	7.60E-38
53	rs12495178	T	C	0.0184	0.6386	0.0017	7.60E-28
54	rs13021737	A	G	-0.0574	0.1681	0.0021	7.50E-157
55	rs17789218	T	C	-0.013	0.7608	0.0019	7.40E-12
56	rs427943	A	C	-0.017	0.4331	0.0017	7.30E-23
57	rs12762034	T	C	-0.024	0.92417	0.0032	7.30E-14
58	rs7196720	T	C	0.0129	0.4932	0.0017	7.30E-14
59	rs657452	A	G	0.0188	0.3784	0.0017	7.20E-29
60	rs4929923	T	C	-0.0181	0.3624	0.0017	7.20E-27
61	rs2481665	T	C	0.0161	0.5592	0.0016	7.20E-23
62	rs6551278	T	C	-0.0181	0.1471	0.0022	7.20E-16
63	rs10824218	A	T	0.0122	0.5522	0.0018	7.20E-12
64	rs40067	A	G	-0.0266	0.1713	0.0023	7.10E-30
65	rs1982441	T	G	0.0175	0.1381	0.0026	7.00E-12
66	rs3902951	T	G	-0.0134	0.7545	0.002	7.00E-12
67	rs11251352	A	G	-0.0109	0.4012	0.0018	7.00E-10
68	rs4981693	A	G	0.0206	0.771	0.002	6.90E-24
69	rs7599312	A	G	-0.0186	0.2652	0.0019	6.90E-24
70	rs4556997	A	C	0.0197	0.1349	0.0024	6.90E-17
71	rs4072917	A	G	0.0115	0.4694	0.0018	6.90E-11
72	rs11577094	T	C	0.0182	0.08109	0.003	6.90E-10
73	rs429343	A	G	0.015	0.4187	0.0017	6.80E-18
74	rs2357760	A	G	0.0145	0.6754	0.0017	6.80E-17
75	rs4660443	T	C	0.0164	0.2218	0.0021	6.80E-15
76	rs4952843	A	G	0.0131	0.6193	0.0018	6.80E-14
77	rs13287131	T	C	-0.0123	0.7509	0.002	6.80E-10
78	rs10182181	A	G	-0.0325	0.5247	0.0016	6.70E-90
79	rs2010281	A	G	-0.0161	0.3552	0.0017	6.70E-21
80	rs11739877	T	C	0.0117	0.6118	0.0018	6.60E-11
81	rs6792696	A	G	0.0104	0.3476	0.0017	6.60E-10
82	rs4500930	T	C	0.0156	0.3461	0.0018	6.50E-18
83	rs7704281	A	G	0.0271	0.04531	0.0041	6.50E-11
84	rs6841761	T	G	-0.0131	0.5252	0.0016	6.40E-16
85	rs11889536	A	G	0.0189	0.8507	0.0024	6.40E-15
86	rs10768994	T	C	0.0114	0.5663	0.0017	6.40E-12

87	rs9927848	A	C	-0.0122	0.7326	0.002	6.40E-10
88	rs7683836	A	G	-0.0114	0.5405	0.0017	6.30E-11
89	rs621042	A	C	-0.0107	0.451	0.0017	6.30E-10
90	rs10962550	C	G	0.0182	0.1801	0.0022	6.20E-16
91	rs10971709	T	C	0.0132	0.2062	0.0021	6.20E-10
92	rs12416812	A	G	0.0111	0.5088	0.0016	6.10E-12
93	rs10968114	A	C	0.0113	0.5319	0.0017	6.10E-11
94	rs17663412	A	C	0.0157	0.1139	0.0027	6.10E-09
95	rs1412235	C	G	0.0246	0.3175	0.0017	6.00E-45
96	rs1431659	A	G	0.0196	0.2656	0.0019	6.00E-24
97	rs1187352	T	C	-0.0119	0.3482	0.0018	6.00E-11
98	rs16849710	A	G	0.0116	0.485	0.0018	6.00E-11
99	rs13191362	A	G	0.0236	0.8802	0.0025	5.90E-21
100	rs3977755	T	C	-0.0135	0.2804	0.0019	5.90E-13
101	rs962796	T	C	0.0148	0.2052	0.0022	5.90E-12
102	rs3829849	T	C	0.0098	0.3589	0.0017	5.90E-09
103	rs156201	C	G	0.0123	0.7606	0.002	5.80E-10
104	rs2832283	A	G	0.0115	0.2208	0.002	5.80E-09
105	rs7615297	C	G	0.0149	0.8535	0.0024	5.70E-10
106	rs7819514	A	G	-0.0107	0.3216	0.0018	5.70E-09
107	rs7498665	A	G	-0.0271	0.5962	0.0017	5.60E-60
108	rs10132280	A	C	-0.0223	0.3017	0.0018	5.60E-35
109	rs4757144	A	G	0.0169	0.5878	0.0018	5.60E-22
110	rs11656076	A	G	-0.0142	0.2254	0.0021	5.60E-12
111	rs17119937	T	C	-0.0212	0.93095	0.0036	5.60E-09
112	rs947612	A	G	-0.0116	0.7516	0.002	5.60E-09
113	rs895330	C	G	0.0201	0.8076	0.0023	5.50E-19
114	rs1503526	T	C	-0.014	0.5162	0.0017	5.50E-17
115	rs200810	T	C	0.0136	0.6284	0.0017	5.50E-16
116	rs9478496	T	C	-0.0152	0.8347	0.0023	5.50E-11
117	rs1928295	T	C	0.0141	0.5539	0.0016	5.40E-18
118	rs756717	A	G	-0.0148	0.3973	0.0017	5.40E-18
119	rs9527895	T	C	-0.0165	0.8371	0.0023	5.40E-13
120	rs774246	A	G	-0.0153	0.8556	0.0025	5.40E-10
121	rs865809	A	G	0.0127	0.2322	0.002	5.40E-10
122	rs11129661	T	C	0.0124	0.2112	0.0021	5.40E-09
123	rs879620	T	C	0.0231	0.6179	0.0018	5.30E-38
124	rs12564992	A	G	-0.0196	0.8856	0.0026	5.30E-14
125	rs7144011	T	G	0.0282	0.2136	0.002	5.20E-47
126	rs1454687	C	G	0.0202	0.4773	0.0017	5.20E-32
127	rs4516268	A	C	-0.0217	0.1925	0.0021	5.20E-25
128	rs12299814	A	C	-0.0157	0.2525	0.002	5.20E-15
129	rs1535660	T	C	0.0147	0.1446	0.0025	5.20E-09
130	rs175165	T	G	0.0103	0.6059	0.0018	5.20E-09

131	rs2007231	T	C	-0.0104	0.6387	0.0018	5.20E-09
132	rs12964689	A	G	0.0203	0.5176	0.0017	5.10E-32
133	rs2281819	A	T	-0.016	0.2302	0.002	5.10E-15
134	rs3800637	T	C	-0.0115	0.664	0.0018	5.10E-10
135	rs11611246	T	G	0.024	0.21	0.002	5.00E-32
136	rs2271189	A	G	-0.0144	0.4051	0.0018	5.00E-16
137	rs11945861	A	G	-0.0148	0.2369	0.002	5.00E-13
138	rs4148155	A	G	0.0188	0.8873	0.0026	5.00E-13
139	rs2051559	T	C	-0.0176	0.8692	0.0026	5.00E-12
140	rs2543132	C	G	0.0146	0.8134	0.0022	5.00E-11
141	rs1296328	A	C	0.0179	0.4343	0.0018	4.90E-24
142	rs7869771	A	C	0.014	0.7353	0.0019	4.90E-13
143	rs2174307	C	G	0.0121	0.4067	0.0017	4.90E-12
144	rs12933482	A	G	-0.0186	0.8952	0.0028	4.90E-11
145	rs6781254	T	C	0.0112	0.3034	0.0018	4.90E-10
146	rs7826312	T	C	-0.0104	0.4121	0.0017	4.90E-10
147	rs11118308	A	G	0.0101	0.5297	0.0016	4.80E-10
148	rs1465900	A	C	0.0125	0.7812	0.002	4.80E-10
149	rs7703576	T	C	-0.0103	0.7115	0.0019	4.80E-08
150	rs11150911	A	C	0.0133	0.2809	0.0018	4.70E-13
151	rs38314	A	G	-0.012	0.4912	0.0017	4.70E-12
152	rs872281	T	C	-0.0151	0.1728	0.0023	4.70E-11
153	rs4589691	C	G	-0.0141	0.8421	0.0024	4.70E-09
154	rs825688	T	C	-0.0095	0.456	0.0017	4.70E-08
155	rs934224	T	C	0.0107	0.7399	0.002	4.70E-08
156	rs9569777	T	G	-0.0192	0.1711	0.0022	4.60E-19
157	rs2228213	A	G	-0.0139	0.3481	0.0017	4.60E-16
158	rs16871902	A	G	0.0125	0.4877	0.0017	4.60E-13
159	rs4801117	A	C	0.0104	0.3676	0.0018	4.60E-09
160	rs7730898	A	G	0.0168	0.729	0.0018	4.50E-20
161	rs1951455	T	C	-0.0145	0.2747	0.0019	4.50E-14
162	rs4653017	T	C	0.0122	0.6818	0.0018	4.50E-11
163	rs12779328	T	C	0.0105	0.2833	0.0019	4.50E-08
164	rs273504	A	G	-0.0153	0.5734	0.0018	4.40E-18
165	rs7925214	T	C	0.0147	0.5133	0.0018	4.40E-17
166	rs8123881	A	G	-0.0196	0.8701	0.0024	4.40E-16
167	rs12680842	A	G	0.0133	0.6795	0.0018	4.40E-14
168	rs6050446	A	G	-0.0343	0.02999	0.0047	4.40E-13
169	rs287104	A	G	0.0115	0.6604	0.0017	4.40E-11
170	rs17425707	T	C	-0.0167	0.8997	0.0028	4.40E-09
171	rs6712	C	G	0.0138	0.1368	0.0025	4.40E-08
172	rs17405819	T	C	0.0215	0.699	0.0018	4.30E-33
173	rs4237643	T	G	0.0223	0.3062	0.0019	4.30E-33
174	rs3904244	A	T	0.0155	0.1377	0.0025	4.30E-10

175	rs16889835	T	C	-0.0145	0.1429	0.0025	4.30E-09
176	rs1445652	A	G	0.0123	0.1855	0.0022	4.30E-08
177	rs2820311	A	G	-0.0235	0.6631	0.0018	4.10E-38
178	rs3803286	A	G	0.0181	0.3431	0.0018	4.10E-23
179	rs2162524	T	C	-0.0155	0.6679	0.0018	4.10E-17
180	rs1436344	C	G	0.0141	0.5922	0.0017	4.10E-16
181	rs12936083	A	G	-0.0139	0.6733	0.0019	4.10E-13
182	rs11736228	A	T	0.0139	0.7413	0.002	4.10E-12
183	rs6500208	A	G	0.014	0.2006	0.002	4.10E-12
184	rs6556301	T	G	-0.0111	0.3596	0.0018	4.10E-10
185	rs7685048	T	C	-0.0101	0.4654	0.0017	4.10E-09
186	rs9288754	T	C	-0.0096	0.4116	0.0017	4.10E-08
187	rs7084454	A	G	0.0193	0.335	0.0019	4.00E-25
188	rs10741329	A	G	0.011	0.681	0.0019	4.00E-09
189	rs1884389	T	C	-0.0103	0.4289	0.0017	4.00E-09
190	rs294704	T	G	-0.0113	0.7239	0.0019	4.00E-09
191	rs9300422	A	G	0.0103	0.3097	0.0018	4.00E-09
192	rs10842166	T	C	0.0109	0.7423	0.002	4.00E-08
193	rs4148866	T	C	0.0098	0.4068	0.0018	4.00E-08
194	rs6772756	A	G	0.0104	0.6628	0.0019	4.00E-08
195	rs2694047	A	G	-0.0188	0.253	0.002	3.90E-21
196	rs10942267	A	G	0.0156	0.6912	0.0019	3.90E-17
197	rs4307239	A	G	-0.0115	0.5422	0.0017	3.90E-11
198	rs1260326	T	C	-0.0105	0.4027	0.0017	3.90E-10
199	rs4430672	T	C	0.0127	0.1996	0.0022	3.90E-09
200	rs7512146	T	G	-0.0094	0.5084	0.0017	3.90E-08
201	rs9382285	A	G	-0.023	0.95387	0.0042	3.90E-08
202	rs1048932	A	C	-0.016	0.4162	0.0017	3.80E-22
203	rs12593036	A	G	0.0154	0.7007	0.0019	3.80E-16
204	rs1150659	A	G	-0.0139	0.2254	0.0019	3.80E-13
205	rs17001561	A	G	0.0151	0.1573	0.0023	3.80E-11
206	rs16822990	A	G	0.0187	0.9199	0.0032	3.80E-09
207	rs3844598	A	G	-0.0095	0.479	0.0017	3.80E-08
208	rs7025938	C	G	-0.0166	0.6813	0.0019	3.70E-19
209	rs2235564	T	C	0.0131	0.3466	0.0018	3.70E-13
210	rs1304070	A	G	0.0126	0.7615	0.002	3.70E-10
211	rs2600226	T	C	-0.0116	0.6697	0.0019	3.70E-10
212	rs11904898	A	G	-0.0121	0.2329	0.0021	3.70E-09
213	rs17513613	T	C	-0.0186	0.6764	0.0018	3.60E-26
214	rs6804842	A	G	-0.0156	0.428	0.0017	3.60E-21
215	rs9989141	T	C	0.0162	0.6387	0.0017	3.60E-21
216	rs2065418	T	G	0.0166	0.6377	0.0018	3.60E-20
217	rs10878946	T	C	-0.0141	0.714	0.0019	3.60E-13
218	rs4722398	T	C	0.0158	0.1336	0.0025	3.60E-10

219	rs380857	A	C	-0.0151	0.8878	0.0027	3.60E-08
220	rs13240600	A	G	0.0204	0.8448	0.0024	3.50E-17
221	rs28350	A	G	0.0177	0.193	0.0022	3.50E-15
222	rs4786903	A	G	-0.0125	0.2632	0.002	3.50E-10
223	rs7313924	C	G	0.0101	0.7138	0.0018	3.50E-08
224	rs10938397	A	G	-0.0324	0.5683	0.0016	3.40E-86
225	rs2124499	C	G	-0.0123	0.3718	0.0017	3.40E-13
226	rs818524	T	C	-0.0106	0.3061	0.0019	3.40E-08
227	rs876605	A	G	0.0108	0.2648	0.002	3.40E-08
228	rs4986044	T	C	-0.0164	0.4687	0.0016	3.30E-23
229	rs4639527	A	G	-0.0172	0.6988	0.0019	3.30E-20
230	rs13184896	T	G	-0.0133	0.4346	0.0016	3.30E-16
231	rs1365466	T	C	-0.0137	0.7406	0.0019	3.30E-13
232	rs2237403	T	C	-0.0121	0.3422	0.0018	3.30E-11
233	rs3754963	A	T	0.0123	0.7426	0.002	3.30E-10
234	rs7222349	A	G	0.0115	0.3441	0.0018	3.30E-10
235	rs10518694	A	C	0.0146	0.1424	0.0025	3.30E-09
236	rs11538	A	G	-0.0135	0.8195	0.0023	3.30E-09
237	rs138289	A	T	0.0103	0.5171	0.0017	3.30E-09
238	rs1982725	T	C	0.0097	0.4778	0.0017	3.30E-08
239	rs806600	A	G	0.0095	0.525	0.0017	3.30E-08
240	rs9783858	T	C	0.0091	0.5191	0.0017	3.30E-08
241	rs7124681	A	C	0.0263	0.4133	0.0016	3.20E-58
242	rs7550711	T	C	0.0649	0.03058	0.005	3.20E-38
243	rs16851483	T	G	0.0369	0.06928	0.0035	3.20E-26
244	rs6443750	T	C	-0.0148	0.1932	0.0021	3.20E-12
245	rs331966	A	C	-0.0112	0.6208	0.0018	3.20E-10
246	rs7933205	A	G	-0.0116	0.2115	0.0021	3.20E-08
247	rs10842240	C	G	0.0218	0.1158	0.0027	3.10E-16
248	rs2246012	T	C	-0.0158	0.8372	0.0022	3.10E-13
249	rs6471941	A	G	0.0156	0.1684	0.0021	3.10E-13
250	rs1521527	C	G	-0.0121	0.5324	0.0017	3.10E-12
251	rs391300	T	C	0.0119	0.3725	0.0017	3.10E-12
252	rs7024334	T	G	0.0138	0.2258	0.002	3.10E-12
253	rs2228552	T	G	0.0127	0.6445	0.0019	3.10E-11
254	rs11781699	T	C	-0.0132	0.8104	0.0021	3.10E-10
255	rs2283093	T	C	0.0127	0.2066	0.0021	3.10E-09
256	rs3764835	A	G	-0.0141	0.1528	0.0024	3.10E-09
257	rs17311369	T	C	-0.0104	0.3278	0.0019	3.10E-08
258	rs11496125	T	C	0.0169	0.4212	0.0017	3.00E-22
259	rs10192119	T	G	-0.0166	0.8327	0.0022	3.00E-14
260	rs765875	T	C	-0.0121	0.4808	0.0017	3.00E-12
261	rs12718572	T	C	-0.0117	0.4024	0.0018	3.00E-11
262	rs13069244	A	G	0.0187	0.07747	0.0032	3.00E-09

263	rs1804528	A	G	0.0109	0.3507	0.002	3.00E-08
264	rs9304665	A	T	0.0229	0.7633	0.002	2.90E-29
265	rs13174863	A	G	-0.0192	0.8452	0.0023	2.90E-16
266	rs1522569	T	G	0.0164	0.8181	0.0022	2.90E-13
267	rs2169642	A	C	-0.0122	0.6313	0.0018	2.90E-11
268	rs13250058	T	G	0.0112	0.6771	0.0018	2.90E-10
269	rs10733051	A	G	0.0097	0.5198	0.0016	2.90E-09
270	rs17446257	A	G	0.0153	0.1292	0.0026	2.90E-09
271	rs4954638	A	C	0.0118	0.7508	0.002	2.90E-09
272	rs7568228	C	G	-0.0102	0.5309	0.0017	2.90E-09
273	rs4482463	A	C	-0.0331	0.9213	0.0033	2.80E-23
274	rs9650755	A	G	-0.0154	0.7336	0.002	2.80E-15
275	rs7503597	T	G	0.0134	0.2934	0.0019	2.80E-12
276	rs7844647	T	C	0.0123	0.7319	0.0018	2.80E-11
277	rs2423668	T	C	0.0105	0.4495	0.0019	2.80E-08
278	rs12939549	A	G	0.018	0.5665	0.0016	2.70E-28
279	rs11505821	A	T	-0.0311	0.9399	0.0035	2.70E-19
280	rs7318817	T	C	-0.0155	0.6071	0.0018	2.70E-18
281	rs13288178	A	G	0.014	0.6406	0.0018	2.70E-15
282	rs10975933	C	G	0.0122	0.6556	0.0018	2.70E-11
283	rs1399471	C	G	-0.0131	0.2608	0.002	2.70E-11
284	rs6011457	A	T	-0.0116	0.4975	0.0017	2.70E-11
285	rs2429150	A	C	-0.0111	0.5836	0.0018	2.70E-10
286	rs8036040	A	C	0.0109	0.4932	0.0017	2.70E-10
287	rs9615905	T	C	0.011	0.45	0.0017	2.70E-10
288	rs10858334	C	G	-0.0143	0.8585	0.0026	2.70E-08
289	rs215634	A	G	0.0152	0.3788	0.0018	2.60E-17
290	rs962273	T	C	-0.0137	0.2943	0.0019	2.60E-13
291	rs12369179	T	C	-0.0359	0.08782	0.0031	2.50E-31
292	rs645040	T	G	0.0171	0.7762	0.002	2.50E-18
293	rs3736485	A	G	0.0134	0.4557	0.0016	2.50E-16
294	rs9522285	A	G	0.0127	0.4143	0.0017	2.50E-13
295	rs1409818	T	C	0.0201	0.1156	0.0029	2.50E-12
296	rs2605603	A	G	-0.0103	0.4887	0.0016	2.50E-10
297	rs9845966	T	G	0.0105	0.4521	0.0017	2.50E-10
298	rs4800191	C	G	0.0103	0.6369	0.0017	2.50E-09
299	rs4906908	T	G	-0.0103	0.4747	0.0017	2.50E-09
300	rs6812882	T	C	-0.0113	0.7288	0.0019	2.50E-09
301	rs7117238	A	G	-0.0131	0.168	0.0022	2.50E-09
302	rs17535749	A	G	0.015	0.1023	0.0027	2.50E-08
303	rs11713193	A	G	0.0239	0.5073	0.0017	2.40E-44
304	rs7102454	T	C	-0.0158	0.6565	0.0018	2.40E-18
305	rs12033257	A	G	0.0146	0.6165	0.0018	2.40E-15
306	rs3807645	A	G	-0.0166	0.221	0.0021	2.40E-15

307	rs559231	T	G	0.0135	0.3956	0.0018	2.40E-14
308	rs326889	T	C	-0.0129	0.3928	0.0018	2.40E-13
309	rs1064213	A	G	0.012	0.492	0.0017	2.40E-12
310	rs9688431	T	C	0.0231	0.93966	0.0035	2.40E-11
311	rs11030618	T	C	0.011	0.5679	0.0017	2.40E-10
312	rs12987009	A	T	-0.011	0.5614	0.0017	2.40E-10
313	rs1896767	A	G	-0.0109	0.5376	0.0017	2.40E-10
314	rs17056301	T	C	-0.0118	0.7364	0.002	2.40E-09
315	rs17424296	A	G	-0.0108	0.3659	0.0018	2.40E-09
316	rs1891216	T	G	-0.0107	0.6241	0.0018	2.40E-09
317	rs2047648	A	T	-0.0118	0.737	0.002	2.40E-09
318	rs4783830	A	G	-0.0105	0.3074	0.0019	2.40E-08
319	rs7138803	A	G	0.03	0.3772	0.0017	2.30E-71
320	rs4740619	T	C	0.0186	0.5479	0.0016	2.30E-30
321	rs769449	A	G	-0.0254	0.1161	0.0027	2.30E-20
322	rs7557796	T	C	0.016	0.3476	0.0018	2.30E-19
323	rs7640424	T	C	-0.0136	0.2969	0.0018	2.30E-14
324	rs7871866	C	G	0.0187	0.1531	0.0024	2.30E-14
325	rs2367112	T	G	0.0119	0.5081	0.0016	2.30E-13
326	rs6448587	A	C	0.0167	0.8109	0.0023	2.30E-13
327	rs4818225	A	G	-0.0117	0.3394	0.0018	2.30E-10
328	rs4671328	T	G	0.0219	0.4467	0.0017	2.20E-36
329	rs7715256	T	G	-0.0166	0.5781	0.0016	2.20E-24
330	rs13047416	C	G	0.0154	0.6231	0.0018	2.20E-17
331	rs13263601	A	C	-0.0154	0.6522	0.0018	2.20E-17
332	rs10510419	T	G	-0.0177	0.1416	0.0023	2.20E-14
333	rs11609659	T	C	0.0154	0.7629	0.002	2.20E-14
334	rs754635	C	G	-0.0198	0.1127	0.0027	2.20E-13
335	rs7334078	T	C	0.0121	0.7118	0.0019	2.20E-10
336	rs902695	A	G	-0.0103	0.4798	0.0017	2.20E-09
337	rs17207196	T	C	-0.0221	0.4118	0.0018	2.10E-35
338	rs4820408	T	G	0.0151	0.408	0.0017	2.10E-19
339	rs17113297	T	C	0.0166	0.2082	0.0021	2.10E-15
340	rs17203016	A	G	-0.015	0.804	0.002	2.10E-13
341	rs1814170	A	T	0.0203	0.8946	0.0029	2.10E-12
342	rs6512302	C	G	0.0142	0.7511	0.002	2.10E-12
343	rs12629015	A	G	0.0135	0.8148	0.0023	2.10E-09
344	rs8071182	A	G	0.0133	0.1735	0.0022	2.10E-09
345	rs592483	T	C	-0.0147	0.5716	0.0017	2.00E-18
346	rs1477887	A	G	-0.0138	0.4567	0.0017	2.00E-15
347	rs2693826	A	G	-0.0137	0.4421	0.0017	2.00E-15
348	rs2163188	C	G	0.0131	0.474	0.0017	2.00E-14
349	rs998732	A	G	0.0171	0.8422	0.0022	2.00E-14
350	rs17238110	A	G	0.0353	0.8366	0.005	2.00E-12

351	rs6595205	C	G	0.0114	0.4695	0.0016	2.00E-12
352	rs10169594	T	C	-0.0121	0.6404	0.0018	2.00E-11
353	rs7941030	T	C	-0.0112	0.6141	0.0017	2.00E-11
354	rs11115176	T	C	0.0121	0.7601	0.0019	2.00E-10
355	rs768840	A	G	0.0114	0.4183	0.0018	2.00E-10
356	rs10248136	T	C	-0.0097	0.5142	0.0017	2.00E-08
357	rs11738695	A	C	0.0097	0.586	0.0017	2.00E-08
358	rs1993709	A	G	-0.0331	0.1823	0.0021	1.90E-57
359	rs1218822	A	G	0.0168	0.6663	0.0017	1.90E-22
360	rs10197031	T	C	-0.0166	0.7166	0.0019	1.90E-18
361	rs901630	T	C	-0.0146	0.3973	0.0017	1.90E-18
362	rs2608703	A	C	0.0142	0.4546	0.0017	1.90E-16
363	rs13110266	A	G	-0.0117	0.4065	0.0017	1.90E-12
364	rs11170468	A	C	0.0123	0.7674	0.0019	1.90E-10
365	rs17551974	A	C	-0.0141	0.1782	0.0022	1.90E-10
366	rs8065336	T	C	-0.012	0.3099	0.0019	1.90E-10
367	rs7761673	A	T	-0.0126	0.2058	0.0021	1.90E-09
368	rs1759975	A	G	0.0096	0.6062	0.0017	1.90E-08
369	rs17767510	C	G	0.0129	0.1697	0.0023	1.90E-08
370	rs6591407	A	C	-0.0118	0.1861	0.0021	1.90E-08
371	rs1927790	T	C	-0.0148	0.5891	0.0016	1.80E-19
372	rs9294260	A	G	0.0147	0.4731	0.0016	1.80E-19
373	rs11084553	A	G	0.021	0.8482	0.0024	1.80E-18
374	rs349088	A	C	-0.0128	0.4976	0.0017	1.80E-13
375	rs2257791	A	G	-0.0142	0.7515	0.002	1.80E-12
376	rs852056	T	C	0.0128	0.2416	0.002	1.80E-10
377	rs12328930	T	C	-0.0098	0.5765	0.0017	1.80E-08
378	rs262130	T	C	0.0127	0.1969	0.0023	1.80E-08
379	rs4713436	T	C	-0.0133	0.1731	0.0024	1.80E-08
380	rs6673081	T	C	0.01	0.4466	0.0018	1.80E-08
381	rs9458814	T	C	-0.0115	0.7668	0.002	1.80E-08
382	rs11672660	T	C	-0.034	0.2049	0.0021	1.70E-60
383	rs12044597	A	G	-0.0143	0.4971	0.0016	1.70E-18
384	rs1327259	A	G	0.0155	0.6128	0.0018	1.70E-18
385	rs7637852	A	G	0.0139	0.3049	0.0019	1.70E-13
386	rs4851029	T	G	-0.0121	0.4753	0.0017	1.70E-12
387	rs10247983	A	G	0.0201	0.9213	0.0033	1.70E-09
388	rs676749	A	T	-0.0104	0.502	0.0017	1.70E-09
389	rs775724	A	G	0.0106	0.4122	0.0018	1.70E-09
390	rs9599161	T	C	0.01	0.5875	0.0017	1.70E-09
391	rs189843	C	G	-0.0098	0.5557	0.0017	1.70E-08
392	rs1999494	A	G	0.0106	0.336	0.0019	1.70E-08
393	rs2307111	T	C	0.0265	0.6038	0.0016	1.60E-58
394	rs9816226	A	T	-0.0323	0.1801	0.0021	1.60E-52

395	rs12150665	T	C	0.0162	0.5942	0.0017	1.60E-22
396	rs663129	A	G	0.0545	0.2301	0.0019	1.60E-178
397	rs6587552	A	G	0.0173	0.2409	0.002	1.60E-17
398	rs3806572	A	G	-0.0145	0.2788	0.0019	1.60E-14
399	rs10887578	C	G	0.0128	0.4896	0.0017	1.60E-13
400	rs2425840	A	C	-0.0119	0.5941	0.0018	1.60E-11
401	rs4858193	T	C	0.0129	0.7221	0.0019	1.60E-11
402	rs10811871	A	G	0.0108	0.6171	0.0018	1.60E-09
403	rs7626079	T	C	0.011	0.3434	0.0018	1.60E-09
404	rs1656377	T	C	-0.0099	0.4115	0.0017	1.60E-08
405	rs17066856	T	C	0.0385	0.90429	0.0028	1.50E-42
406	rs17806379	T	C	-0.0258	0.1789	0.0022	1.50E-30
407	rs6985109	A	G	-0.0177	0.5338	0.0017	1.50E-26
408	rs10920678	A	G	0.0155	0.4291	0.0016	1.50E-21
409	rs6235	C	G	-0.0175	0.7298	0.0019	1.50E-19
410	rs2479958	A	G	0.0154	0.4925	0.0018	1.50E-17
411	rs4864201	T	C	0.0141	0.3531	0.0017	1.50E-16
412	rs17327461	T	C	0.0125	0.4498	0.0016	1.50E-14
413	rs1330052	C	G	-0.0132	0.6496	0.0018	1.50E-13
414	rs16953563	A	G	-0.0134	0.2516	0.002	1.50E-11
415	rs2903971	T	G	0.0145	0.8203	0.0023	1.50E-10
416	rs11773362	T	C	-0.0111	0.3369	0.0018	1.50E-09
417	rs4358081	A	C	-0.0097	0.5369	0.0017	1.50E-08
418	rs2744974	T	C	0.0249	0.338	0.0018	1.40E-45
419	rs11165643	T	C	0.0206	0.5828	0.0017	1.40E-35
420	rs208015	T	C	0.0356	0.0784	0.0034	1.40E-25
421	rs3800229	T	G	0.0175	0.7123	0.0018	1.40E-22
422	rs355777	C	G	0.0153	0.4106	0.0017	1.40E-18
423	rs8192675	T	C	-0.0152	0.7112	0.0018	1.40E-17
424	rs7535528	A	G	-0.0152	0.3741	0.0018	1.40E-16
425	rs10269783	A	G	0.0133	0.3896	0.0017	1.40E-15
426	rs2885415	A	G	-0.0158	0.2531	0.002	1.40E-15
427	rs9806742	A	G	0.0208	0.8826	0.0026	1.40E-15
428	rs9951619	T	G	-0.0156	0.2357	0.002	1.40E-15
429	rs12527426	A	G	0.0135	0.2969	0.0019	1.40E-12
430	rs4414033	A	G	0.0129	0.627	0.0018	1.40E-12
431	rs4936175	T	C	-0.0122	0.5555	0.0017	1.40E-12
432	rs7313220	A	G	0.0122	0.4861	0.0017	1.40E-12
433	rs12905439	C	G	0.0118	0.6607	0.0018	1.40E-10
434	rs1863652	A	G	-0.0115	0.3449	0.0018	1.40E-10
435	rs6764533	A	G	0.0116	0.359	0.0018	1.40E-10
436	rs1830074	T	C	-0.0115	0.712	0.0019	1.40E-09
437	rs2284746	C	G	0.0104	0.4762	0.0017	1.40E-09
438	rs629443	T	G	0.0116	0.2501	0.0019	1.40E-09

439	rs8027205	C	G	0.0108	0.6033	0.0018	1.40E-09
440	rs1372177	C	G	-0.0098	0.5194	0.0017	1.40E-08
441	rs2931434	T	C	-0.0104	0.3168	0.0018	1.40E-08
442	rs4151664	T	C	0.02	0.05847	0.0035	1.40E-08
443	rs999889	A	G	-0.0108	0.2818	0.0019	1.40E-08
444	rs889398	T	C	-0.0196	0.4247	0.0016	1.30E-32
445	rs1884897	A	G	-0.0194	0.3692	0.0017	1.30E-30
446	rs2365389	T	C	-0.0174	0.4143	0.0017	1.30E-25
447	rs977747	T	G	0.0169	0.4051	0.0017	1.30E-24
448	rs7903146	T	C	-0.0181	0.2912	0.0018	1.30E-23
449	rs2861683	A	C	0.0144	0.593	0.0017	1.30E-16
450	rs1452075	T	C	0.0141	0.7277	0.0018	1.30E-14
451	rs10832778	C	G	-0.0125	0.3778	0.0017	1.30E-13
452	rs217671	A	G	-0.0144	0.7281	0.0019	1.30E-13
453	rs2196618	A	G	-0.0147	0.2754	0.002	1.30E-13
454	rs2791653	A	G	0.0141	0.2423	0.0019	1.30E-13
455	rs2907948	A	G	-0.0141	0.2427	0.0019	1.30E-13
456	rs538579	C	G	0.0137	0.3228	0.0019	1.30E-13
457	rs7206608	C	G	-0.0132	0.6854	0.0019	1.30E-12
458	rs845084	A	G	0.014	0.2678	0.002	1.30E-12
459	rs10915840	A	G	-0.0118	0.283	0.0019	1.30E-09
460	rs12675063	A	T	-0.0156	0.8869	0.0026	1.30E-09
461	rs1583123	A	C	0.0108	0.6224	0.0018	1.30E-09
462	rs2836964	T	C	0.011	0.6424	0.0018	1.30E-09
463	rs9408882	A	G	-0.0093	0.4594	0.0016	1.30E-08
464	rs17636031	T	C	-0.016	0.7299	0.0019	1.20E-17
465	rs9827823	T	C	0.0193	0.8511	0.0024	1.20E-15
466	rs1158805	A	C	-0.0137	0.3766	0.0018	1.20E-14
467	rs1075901	T	C	-0.0121	0.4361	0.0016	1.20E-13
468	rs6461115	A	G	0.0144	0.7715	0.0019	1.20E-13
469	rs543874	A	G	-0.0475	0.8048	0.002	1.20E-122
470	rs339991	A	G	-0.0124	0.4369	0.0018	1.20E-12
471	rs1188017	T	C	-0.0142	0.1954	0.0021	1.20E-11
472	rs2875762	C	G	0.0139	0.2473	0.002	1.20E-11
473	rs7173441	A	T	0.0119	0.402	0.0018	1.20E-11
474	rs9362662	A	G	0.0112	0.4799	0.0017	1.20E-10
475	rs10747488	A	C	-0.0123	0.7601	0.002	1.20E-09
476	rs13107325	T	C	0.047	0.07373	0.0032	1.10E-47
477	rs3814883	T	C	0.0232	0.4764	0.0017	1.10E-40
478	rs1579557	T	C	0.0213	0.2998	0.0019	1.10E-29
479	rs7788008	A	G	-0.0157	0.4445	0.0017	1.10E-19
480	rs3007105	T	C	0.0142	0.4697	0.0017	1.10E-17
481	rs6692586	A	G	0.0192	0.168	0.0023	1.10E-16
482	rs12364470	T	G	-0.0178	0.8374	0.0022	1.10E-15

483	rs12981256	A	G	0.0142	0.5325	0.0018	1.10E-15
484	rs946824	T	C	0.0206	0.141	0.0026	1.10E-15
485	rs1948080	T	G	0.0137	0.6251	0.0018	1.10E-14
486	rs10742752	T	C	-0.0124	0.3841	0.0017	1.10E-13
487	rs11951673	T	C	-0.0123	0.3941	0.0017	1.10E-13
488	rs2143253	A	G	-0.0188	0.1189	0.0026	1.10E-12
489	rs17014375	T	G	-0.0172	0.8652	0.0025	1.10E-11
490	rs4880341	T	C	-0.0118	0.5606	0.0017	1.10E-11
491	rs7172627	A	G	-0.0117	0.5281	0.0017	1.10E-11
492	rs11105839	A	T	-0.0109	0.3799	0.0017	1.10E-10
493	rs10984756	C	G	-0.0174	0.8952	0.0029	1.10E-09
494	rs11173522	A	C	0.0128	0.2078	0.0021	1.10E-09
495	rs3732084	T	C	-0.0107	0.3861	0.0018	1.10E-09
496	rs491711	A	C	0.0115	0.684	0.0019	1.10E-09
497	rs1241986	A	G	-0.0139	0.8479	0.0024	1.10E-08
498	rs1624134	C	G	0.0101	0.4068	0.0018	1.10E-08
499	rs17499593	C	G	-0.0125	0.8103	0.0022	1.10E-08
500	rs6265	T	C	-0.0412	0.1951	0.0021	1.00E-86
501	rs13329567	T	C	-0.0293	0.2308	0.002	1.00E-50
502	rs11880870	A	G	0.0189	0.5199	0.0017	1.00E-28
503	rs12049202	T	C	0.024	0.203	0.0022	1.00E-28
504	rs17724992	A	G	0.0183	0.7404	0.0019	1.00E-22
505	rs7206790	C	G	-0.0627	0.5293	0.0017	1.00E-200
506	rs7181498	T	C	0.0163	0.3691	0.0018	1.00E-19
507	rs930295	A	C	0.0211	0.1583	0.0023	1.00E-19
508	rs10953740	A	G	0.0153	0.4466	0.0017	1.00E-18
509	rs7899106	A	G	-0.0331	0.95223	0.0037	1.00E-18
510	rs7780752	T	C	-0.0139	0.64	0.0018	1.00E-14
511	rs1421334	A	C	0.0125	0.4569	0.0018	1.00E-12
512	rs17710386	T	C	-0.0126	0.6681	0.0018	1.00E-12
513	rs12922346	C	G	0.0136	0.2657	0.002	1.00E-11
514	rs9367368	T	C	0.0121	0.6967	0.0018	1.00E-11
515	rs12881629	A	G	-0.0212	0.92054	0.0033	1.00E-10
516	rs1268065	A	G	-0.0102	0.4794	0.0017	1.00E-09
517	rs4518345	A	G	-0.0117	0.2842	0.0019	1.00E-09
518	rs6734537	T	C	-0.0133	0.8029	0.0022	1.00E-09
519	rs13147390	T	C	-0.0103	0.6431	0.0018	1.00E-08
520	rs1492767	T	C	0.0094	0.4957	0.0016	1.00E-08
521	rs1885728	A	G	0.0108	0.6787	0.0019	1.00E-08

SNP:Single nucleotide polymorphism; EA: effect allele; OA: other allele; EAF: expected average frequency; SE: standard error.

Supplementary Table 5. Univariate Mendelian randomization heterogeneity test

Exposures	Outcomes	Methods	Cochran's Q statistic	Q-pval
Smoking	NAFLD	MR Egger	114.17	0.478
Smoking	NAFLD	IVW	122.39	0.301
BMI	NAFLD	MR Egger	601.35	0.001
BMI	NAFLD	IVW	601.56	0.001
Smoking	BMI	MR Egger	1261.94	<0.001
Smoking	BMI	IVW	1278.14	<0.001

BMI, body mass index; NAFLD, non-alcoholic fatty liver disease; IVW, inverse variance weighted; MR, Mendelian randomization

Supplementary Table 6. MR-Egger test for pleiotropy

Exposures	Outcomes	MR-Egger intercept	SE	pval
Smoking	NAFLD	0.02	0.007	0.496
BMI	NAFLD	0.001	0.003	0.677
Smoking	BMI	-0.001	0.005	0.813

BMI, body mass index; NAFLD, non-alcoholic fatty liver disease; MR, Mendelian randomization; SE, standard error.

Supplementary Table 7. MR-PRESSO test for pleiotropy

Exposures	Outcomes	Outlier	Global Test-pvalue	Outlier-corrected Estimate	Outlier-corrected pvalue
Smoking	NAFLD	NA	0.332	0.562*	<0.001*
BMI	NAFLD	3	0.559	0.455	<0.001
Smoking	BMI	2	0.246	0.475	<0.001

*, Shows the raw MR-PRESSO results, since there are no outliers and the MR-PRESSO test is not tested for correction of outliers; BMI, body mass index; NAFLD, non-alcoholic fatty liver disease; MR, Mendelian randomization;

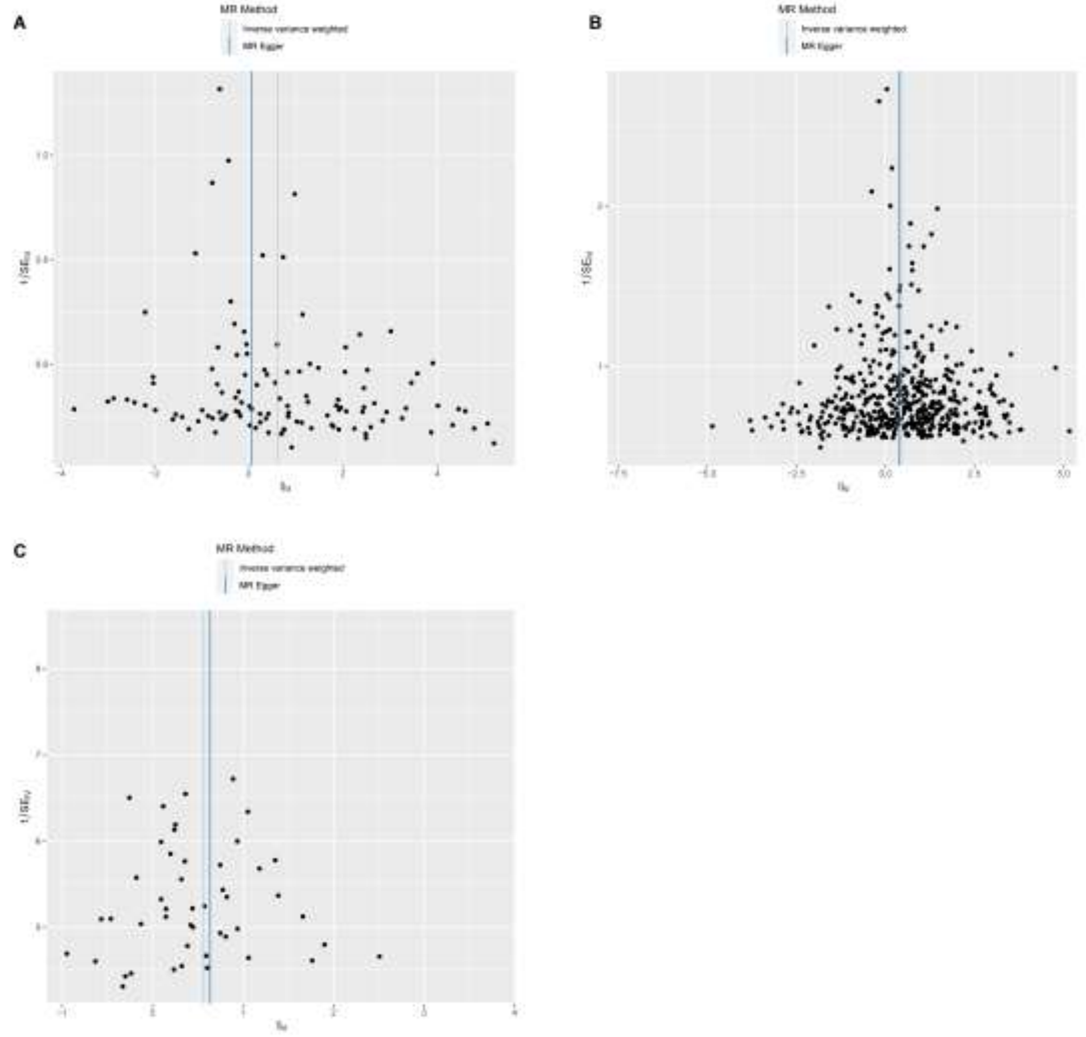

Supplemental File 8. Funnel plot

A: Smoking on non-alcoholic fatty liver disease; B: body mass index on non-alcoholic fatty liver disease; C: Smoking on body mass index.

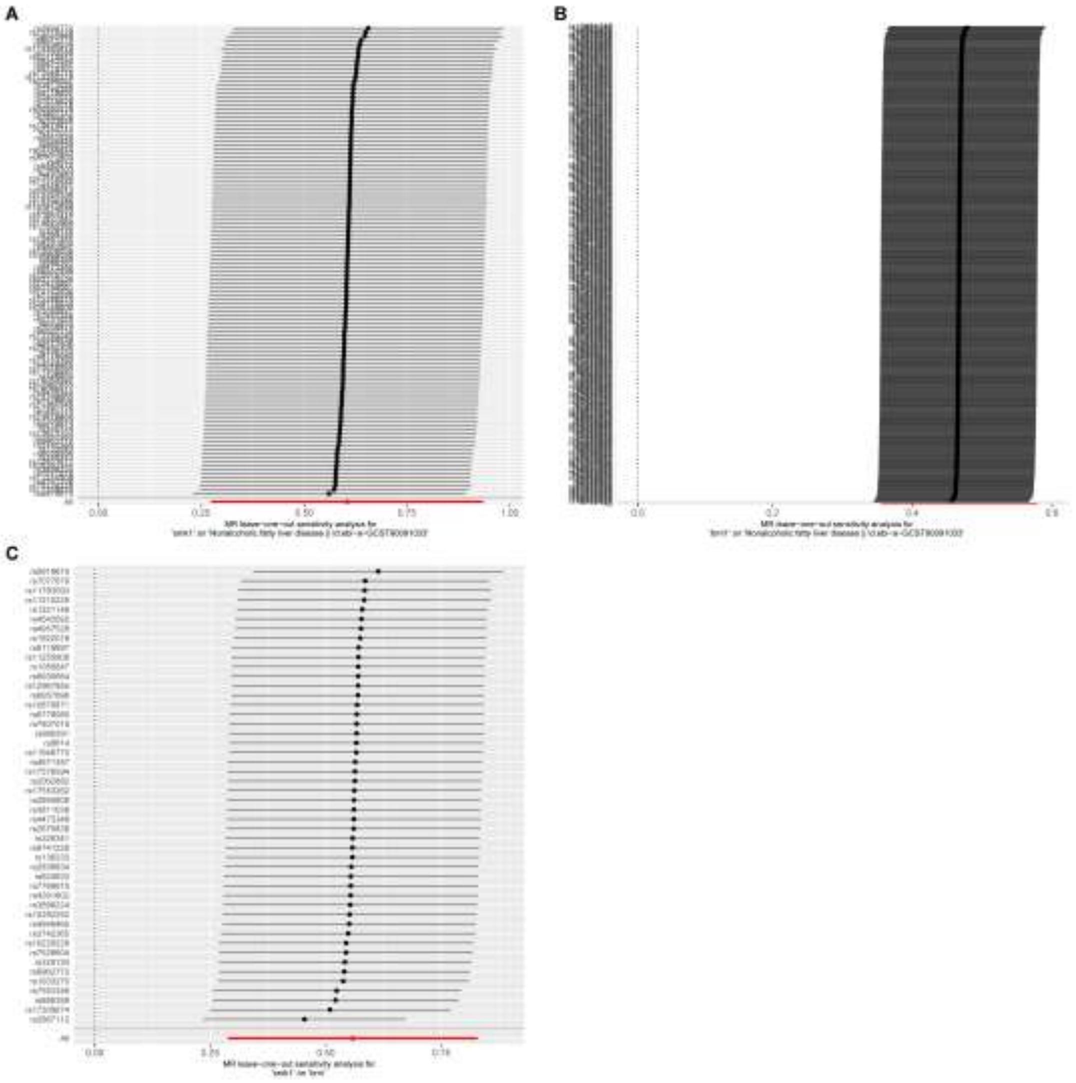

Supplemental File 9. Leave-one-out plot

A: Smoking on non-alcoholic fatty liver disease; B: body mass index on non-alcoholic fatty liver disease; C: Smoking on body mass index.

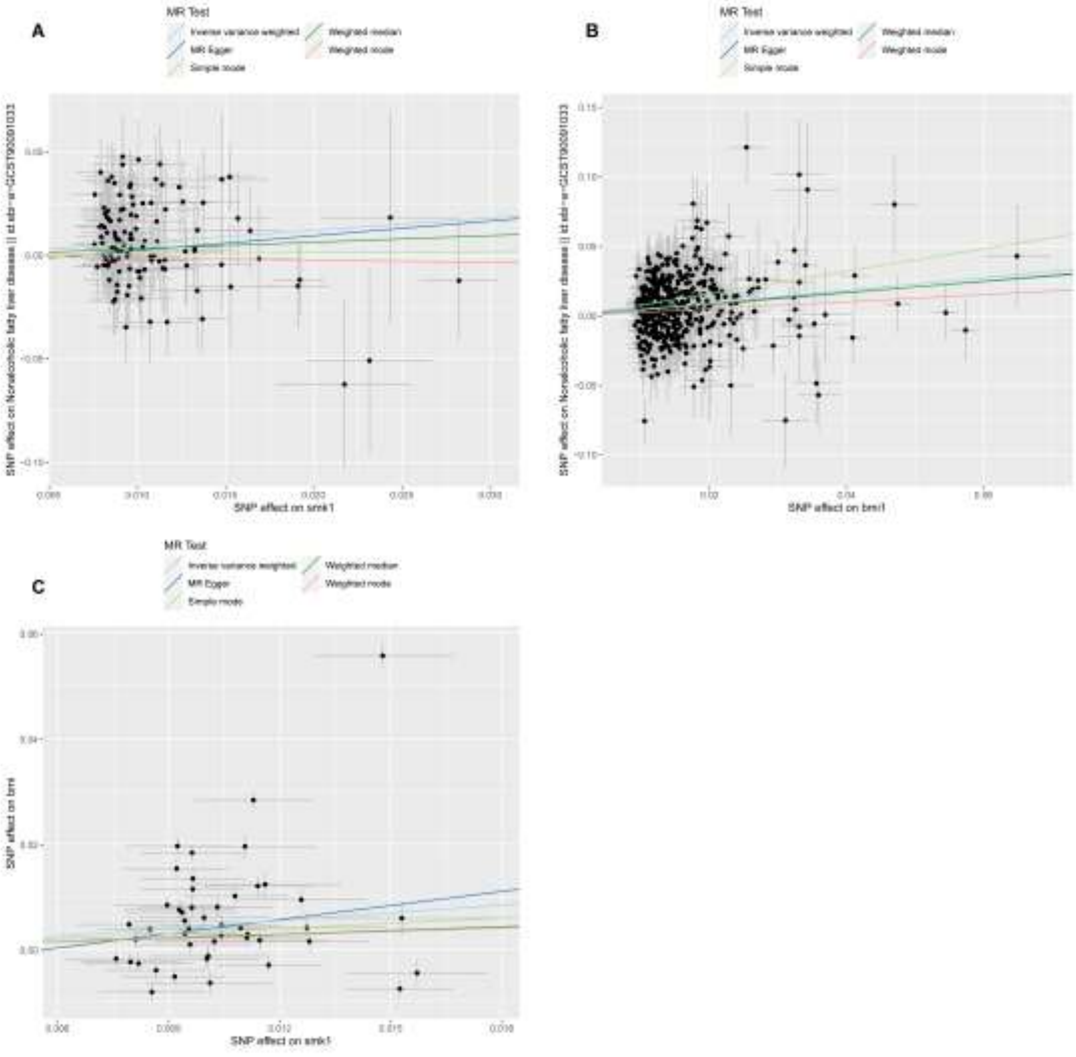

Supplemental File 10 A: Smoking on non-alcoholic fatty liver disease; B: body mass index on non-alcoholic fatty liver disease; C: Smoking on body mass index.
